# Supplementary material for: Clinical Prognosis of Lung Cancer in Patients with Moderate Chronic Kidney Disease
Source: Cancers (Basel). 2022 Sep 30;14(19):4786. doi: 10.3390/cancers14194786 (PMC9562850; doi:10.3390/cancers14194786)
Supplement: Supplementary file 1 [file cancers-14-04786-s001.zip › cancers-1912322-supplementary.pdf]

Supplementary Table S1. Selected treatment options for lung cancer according to CKD stage in early and advanced lung cancer stage.

		Stage I–IIIA		Stage IIIB–IV		*p*-value
		N	%	N	%	
CKD 3 (n=117)		44	100	73	100	
Treatment options	Surgery	28	63.6	3	4.1	< 0.001
	Adjuvant Chemotherapy	11				
	Chemotherapy	2	4.5	27	37.0	
	CCRT	4	9.1	8	11.0	
	Radiotherapy	2	4.5	3	5.5	
	Supportive care	6	13.6	30	42.5	
5-year survival		11	25.0	1	1.4	< 0.001
CKD 4 (n=42)		17	100	25	100	
Treatment options	Surgery	8	47.1	0	0.0	< 0.001
	Adjuvant Chemotherapy	2				
	Chemotherapy	0	0.0	11	44.0	
	CCRT	0	0.0	2	8.0	
	Radiotherapy	2	17.6	3	16.0	
	Supportive care	6	35.3	8	32.0	
5-year survival		1	5.9	1	4.0	0.779
CKD 5 (n=22)		9	100	13	100	
Treatment options	Surgery	6	66.7	0	0.0	0.002
	Adjuvant Chemotherapy	0				
	Chemotherapy	0	0.0	5	38.5	
	CCRT	1	11.1	1	7.7	
	Radiotherapy	2	22.2	1	7.7	
	Supportive care	0	0.0	6	46.2	
5-year survival		2	22.2	1	7.7	0.329

Supplementary Table S2. Clinical outcomes of lung cancer patients with chronic kidney disease according to treatment options.

	All patients		CKD 3		CKD 4		CKD 5	
	OS	PFS	OS	PFS	OS	PFS	OS	PFS
Palliative therapy	3.2 (1.0-8.2)	-	3.5(1.0-8.4)	-	3.5 (1.1-8.2)	-	1.1 (0.3-3.2)	-
Surgery								
Pneumonectomy	30.1	-	30.1	-	-	-		-
Lobectomy	45.9 (24.3-69.2)	-	48.1 (27.9-71.5)	-	3.5 (1.1-8.2)	-	33.4 (4.8-64.9)	-
Wedge resection	53.5 (11.5-90.9)	-	72.2 (53.5-90.9)	-	-	-	11.53	-
Adjuvant therapy		-		-		-		-
Yes	40.4 (29.3-50.0)	-	45.9 (30.6-50.0)	-	25.4 (10.4-40.3)	-	-	-
No	50.9 (21.1-85.1)	-	59.3 (27.0-98.7)	-	40.3 (11.6-66.6)	-	22.5 (5.0-63.6)	-
Radiotherapy								
Radiotherapy only	16.9 (3.8-57.0)	-	30.6 (7.9-58.8)	-	6.0 (2.8-19.0)	-	49.3 (41.2-57.4)	-
Combined chemotherapy	16.1 (7.4-29.9)	4.6 (2.9-12.1)	11.1 (6.2-19.7)	4.1 (2.8-9.5)	12.4 (1.0-23.8)	5.3 (4.9-5.6)	39.7 (11.5-60.9)	19.0 (2.8-56.3)
Chemotherapy								
1st chemotherapy	10.5 (4.5-22.3)	4.6 (2.8-10.9)	10.7 (6.3-21.0)	4.7 (2.9-7.8)	4.0 (1.3-24.6)	4.9 (0.58-10.6)	11.5 (4.5-36.5)	4.4 (2.6-17.3)
AP	10.7 (7.3-16.5)	3.9 (3.1-12.4)	12.0 (8.4-16.2)	3.9 (3.1-12.4)	-	-	-	-
GP	13.8 (8.0-23.4)	5.1 (4.0-7.0)	10.7 (9.1-18.2)	5.5 (4.5-8.2)	-	-	8.0 (4.5-11.5)	3.4 (2.8-4.0)
GC	13.8 (7.9-23.3)	5.1 (1.3-15.0)	18.4 (11.5-26.6)	5.1 (1.69-19.2)	6.6 (0.7-12.5)	6.4 (0.4-12.3)	-	-

TP	21.7 (6.7-49.3)	7.5 (2.7-16.3)	20.5 (5.4-24.0)	7.1 (2.6-12.3)	122.0	16.1	-	-
TC	5.2 (1.7-9.7)	3.8 (1.5-9.9)	16.2 (4.75-60.0)	4.1 (3.4-11.9)	2.4	0.8	-	-
EC	5.2 (1.8-9.0)	4.2 (1.5-6.0)	6.9 (2.5-9.7)	5.3 (4.1-7.7)	1.7 (0.8-1.8)	1.1 (0.5-2.1)	-	-
EP	16.5 (10.5-27.1)	5.4 (3.4-15.7)	4.4 (4.2-12.3)	3.4 (0.8-5.4)	12.4 (1.0-23.8)	5.3 (4.9-5.6)	33.1 (25.6-39.7)	17.3 (15.7-19.0)
2nd chemotherapy	18.8 (12.0-27.6)	-	15.1 (10.4-19.7)	-	32.1 (15.1-101.6)	-	30.0 (10.0-54.0)	-
3rd chemotherapy	18.2 (9.4-30.1)	-	17.4 (10.9-21.8)	-	40.3	-	30.0 (26.6-33.4)	-
Targeted therapy	18.2 (9.5-30.8)	10.4 (3.3-18.7)	8.0 (1.8-16.6)	4.7 (28-12.9)	26.9 (12.8-98.6)	10.6 (7.4-20.0)	4.8 (4.5-47.1)	19.1 (4.6-54.0)
Gefitinib	20.5 (16.9-54.0)	12.4 (3.8-25.2)	18.5 (16.9-21.4)	14.7 (2.2-14.8)	122.0	-	33.4 (4.8-60.9)	10.1 (4.4-56.3)
Erlotinib	10.5 (8.7-24.8)	20.0	10.5 (9.8-19.3)	-	28.3	20.0	26.57	-
afatinib	39.5 (25.5-58.2)	10.6 (2.9-27.0)	48.9 (39.5-58.2)	15.0 (.9-27.0)	25.5	10.6	-	-

Data are presented as the median value (interquartile range) or number. CKD, chronic kidney disease; OS, overall survival; PFS, progression free survival; AP, pemetrexed with cisplatin; GP, gemcitabine with cisplatin; GC, gemcitabine with carboplatin; TP, paclitaxel with cisplatin; TC, paclitaxel with carboplatin; EC, etoposide with carboplatin; EP, etoposide with cisplatin
